# Young people’s perceptions of smartphone-enabled self-testing and online care for sexually transmitted infections: qualitative interview study

**DOI:** 10.1186/s12889-016-3648-y

**Published:** 2016-09-13

**Authors:** Catherine R. H. Aicken, Sebastian S. Fuller, Lorna J. Sutcliffe, Claudia S. Estcourt, Voula Gkatzidou, Pippa Oakeshott, Kate Hone, S. Tariq Sadiq, Pam Sonnenberg, Maryam Shahmanesh

**Affiliations:** 1 Research Department of Infection and Population Health, University College London, Mortimer Market Centre, off Capper Street, London, WC1E 6JB UK; 2 Institute for Infection and Immunity, St George’s University of London, London, UK; 3 Blizard Institute, Centre for Immunology and Infectious Diseases, Barts and the London School of Medicine and Dentistry, Queen Mary University of London, London, UK; 4 College of Engineering, Design and Physical Sciences, Brunel University London, London, UK; 5 Population Health Research Institute, St George’s University of London, London, UK

**Keywords:** Acceptability of healthcare, Clinical pathways, eHealth, Internet, Mobile health, Sexually transmitted infections

## Abstract

**Background:**

Control of sexually transmitted infections (STI) is a global public health priority. Despite the UK’s free, confidential sexual health clinical services, those at greatest risk of STIs, including young people, report barriers to use. These include: embarrassment regarding face-to-face consultations; the time-commitment needed to attend clinic; privacy concerns (e.g. being seen attending clinic); and issues related to confidentiality.

A smartphone-enabled STI self-testing device, linked with online clinical care pathways for treatment, partner notification, and disease surveillance, is being developed by the *e*STI^2^ consortium. It is intended to benefit public health, and could do so by increasing testing among populations which underutilise existing services and/or by enabling rapid provision of effective treatment. We explored its acceptability among potential users.

**Methods:**

In-depth interviews were conducted in 2012 with 25 sexually-experienced 16–24 year olds, recruited from Further Education colleges in an urban, high STI prevalence area. Thematic analysis was undertaken.

**Results:**

Nine females and 16 males participated. 21 self-defined as Black; three, mixed ethnicity; and one, Muslim/Asian. 22 reported experience of STI testing, two reported previous STI diagnoses, and all had owned smartphones.

Participants expressed enthusiasm about the proposed service, and suggested that they and their peers would use it and test more often if it were available. Utilizing sexual healthcare was perceived to be easier and faster with STI self-testing and online clinical care, which facilitated concealment of STI testing from peers/family, and avoided embarrassing face-to-face consultations. Despite these perceived advantages to privacy, new privacy concerns arose regarding communications technology: principally the risk inherent in having evidence of STI testing or diagnosis visible or retrievable on their phone. Some concerns arose regarding the proposed self-test’s accuracy, related to self-operation and the technology’s novelty. Several expressed anxiety around the possibility of being diagnosed and treated without any contact with healthcare professionals.

**Conclusions:**

Remote STI self-testing and online care appealed to these young people. It addressed barriers they associated with conventional STI services, thus may benefit public health through earlier detection and treatment. Our findings underpin development of online care pathways, as part of ongoing research to create this complex e-health intervention.

**Electronic supplementary material:**

The online version of this article (doi:10.1186/s12889-016-3648-y) contains supplementary material, which is available to authorized users.

## Background

Sexually transmitted infections (STI) are a major public health issue in England, and young people are particularly affected [1, 2]. STI services seek to identify, diagnose and treat people with these often asymptomatic infections, in order to prevent transmission and minimise medical complications associated with repeat and long-term infection.

Young people’s high STI rates persist despite good provision of confidential, free sexual healthcare in the UK (including London, where our study took place), through a range of specialist and community services, and the National Chlamydia Screening Programme (NCSP) for England’s sexually-active under-25s [1, 2]. Specialist genito-urinary medicine (GUM) clinics provide free testing and treatment for a comprehensive range of STIs and HIV. Sexual health clinics specifically for young people (e.g. Brook) provide free contraceptive and sexual health services, with STI service provision varying between clinics. STI testing is often available through contraceptive clinics and general practice, also without charge. Within the NCSP, free screening for chlamydia is delivered through various channels, including community and healthcare settings, and via internet-ordered postal home-sampling kits (a service which was widely-available in the years leading up to our study [3]). Self-taken samples of urine (for males) or vulvo-vaginal swabs (for females) are sent to a laboratory for testing with the result communicated some days after, typically by telephone or text message (SMS).

Over the last decade there have been considerable efforts to widen access to sexual health services by extended and weekend opening hours within specialist services, delivered from National Health Service (NHS) genitourinary medicine (GUM)/sexual health clinics. These are open-access (can be used without referral), offer comprehensive STI testing and account for the majority of reported STI diagnoses [2]. However, STI clinic attendance is viewed by some as stigmatising [4], which negatively impacts upon expectations and experiences of attending clinic [5]. General practitioners (GPs) have been encouraged to take on greater roles in sexual health but have been perceived to offer variable quality, less confidential services [4, 6]. Across all settings, young people report fear of judgment by staff, and embarrassment, which can deter sexual healthcare-seeking, with particular concerns regarding face-to-face consultations [6]. Young people also report embarrassment [7] and stigma [8] associated with accepting offers of chlamydia screening even though this does not require a consultation. Receiving chlamydia home-sampling kits by post avoids face-to-face offers of screening, but can compromise privacy [4].

Currently, reliable rapid point of care tests for many STIs are unavailable, but could deliver benefits in terms of reduced complications, from faster provision of treatment in clinics [9, 10], and reduced transmission, since sexual risk behaviour may continue while patients are awaiting diagnosis and treatment [11, 12]. Such benefits may also be derived from a rapid self-test, provided that users who test positive are promptly and effectively treated. The *Electronic Self-testing Instruments for Sexually Transmitted Infections* (*e*STI^2^) consortium is developing an accurate, rapid smartphone-enabled diagnostic self-test for multiple STIs, linked to online clinical management pathways which would be designed to provide safe, appropriate treatment and care. This complex intervention makes use of young adults’ relatively high use of internet [13] and smartphone technologies [14]. It is envisaged that users would register (providing information for public health surveillance), do the test, receive their diagnosis and, if positive, provide medical information to enable safe prescribing of appropriate antibiotic treatment, all online via their smartphone. If appropriate, antibiotic treatment could be posted to them, or an electronic authorisation (“e-prescription”) could allow collection from a pharmacy. For many users, this whole process could take place ‘remotely’, without seeing or speaking to healthcare professionals, or attending clinical settings.

Smartphone-enabled STI self-testing, linked to online clinical management pathways, is a unique and complex intervention. Although some examples of online STI care exist [3, 15–21] these only represent parts of the remote online care pathway we propose, with limited information on acceptability. Qualitative research on the acceptability of home self-testing [22] and internet use in relation to STI testing [23–26] suggests that potential users have reservations around safety, test reliability, online privacy and confidentiality. Much of this research [22–25] was conducted in the US and Canada (i.e. differing health service contexts), and findings may not be transferable to our proposed intervention. Therefore, formative research was needed to inform the development of our proposed complex e-health intervention [27–29], which is intended as an adjunct to existing services (rather than a replacement) and which may reach populations which under-use existing services.

In this study, we explored perceptions and acceptability of remote STI self-testing and associated online care pathways to treatment (a hypothetical intervention), among young people from an Inner-London locality with high rates of STIs [30] and large populations of Black Caribbean and African ethnic origin. As our study population reflects public health need for STI services, and young urban populations may also be ‘early adopters’ of new technologies, we considered them potential users of our novel intervention.

This study took place early in the development of the self-test and the online care pathways, and was part of a programme of formative research which informed this complex intervention’s development. Other formative research considered user-interface design [31] and clinical care quality and safety [32], which together with this study led to a recent proof-of-concept study of an online care pathway for chlamydia, with mixed-methods evaluation. Survey research has provided indicative evidence about the user population [33], and development of the self-testing device [34] is ongoing.

## Methods

### Study design and population

Individual in-depth interviews were conducted with young people, recruited from an Inner-London Further Education (FE) college. In the UK, FE colleges provide post-compulsory education for those aged 16 and older, often vocational, and are distinct from Higher Education institutions – universities – which provide degree-level academic qualifications. People from lower socio-economic groups are over-represented among FE college students. Eligible students were aged 16–24 years, and self-reported having had sex at least once.

### Sampling and recruitment

A purposive sampling strategy [35] was used, with gender and age-group as primary sampling criteria, and a target of 24–36 interviews. We used the age-groups 16–19 and 20–24 because experience with sex, and with sexual healthcare and healthcare in general, are likely to increase with increasing age. Furthermore these categories are similar to those used in national STI surveillance data. Following an email sent on behalf of the researchers to all students, and posters placed in the college, students were approached in college communal areas by the interviewer, or referred to him by staff. The interviewer explained to potential participants that the study would involve a face-to-face interview with him, which would last about an hour, to find out what they thought about a new way of testing for STIs. Further details of the study were provided orally and in information sheets.

### Procedure

Interviews took place in private rooms at college sites. One male interviewer (SF) conducted and audio-recorded all interviews. The topic guide, described briefly here, had been piloted, and was used flexibly and revised iteratively between interviews. The interviewer began by asking about participants’ experience with smartphone technology, internet-use in relation to health, and STI testing. First impressions of ‘testing for STIs using your smartphone’ were explored. Then, participants were provided with a brief description of the proposed testing device and associated online care pathway, aided by an animation (Additional file 1) which outlined stages a user would potentially go through (operating the self-testing device with a sample of urine or vaginal swab, receiving their result, and if positive, an online consultation, ‘e-prescription’, partner notification and sexual health advice). The interviewer explained that the test was still being developed, but that the animation showed what it might be like. Few details were provided about the test and online care pathway, for simplicity, and because of uncertainties at this stage in intervention development. The interviewer explained that obtaining treatment this way would be safe for most people (but not what would happen otherwise). Scenarios were used to explore acceptability and preferences of various stages, from self-testing, through to receipt of treatment for those testing positive (Additional file 2). Participants were asked for their understanding of ‘confidentiality’. Interviews explored acceptability of providing personal details, sexual history, and medical information to verify treatment safety, using their smartphone. Participants were asked if they would use the service described and why (not). The interviewer, mindful of his somewhat older age, status as a university researcher, association with novel technology, and the implications of these for social desirability bias in the views participants might express, sought to lessen the social distance between himself and participants by mirroring participants’ language use, and emphasised that he was not developing the intervention and so would not be offended if they did not like or agree with some or all of the proposed format. The interviewer kept field-notes, recording circumstances of recruitment and impressions from interviews. Interviews lasted 29–75 min (mean: 53mins). Each participant received £15 in recognition of their time and contribution to the study.

Interviews focused on exploring novel aspects of the proposed intervention; aspects that are established as broadly acceptable or have become common practice (e.g. self-sampling [36], receipt of STI test results by text-message) were not explored. Details unknown at the time of the interviews were also not explored unless mentioned by interviewees (including: which infections the device would detect – described by the interviewer as chlamydia in the first instance ‘because it is an easier infection to treat’, specific clinical and disease surveillance information to be collected, cost, distribution, and whether the device would be for single or repeat use). These are being explored in ongoing research.

### Analysis

A thematic analysis [37] was conducted by CA, using NVivo software and paper charts. For data familiarisation, transcripts were read repeatedly, alongside listening to recordings and reading field-notes. A mixed inductive-deductive approach was used: identification of themes was influenced by emergent and recurring issues in the data, and by *a priori* issues relating to study aims. Individuals’ accounts of their views and experiences with existing STI testing services, and with smartphones and the internet, were used to contextualise their views on the novel service. Analysis took place after data collection was complete, meaning that initial findings could not be explored in subsequent interviews.

SF and MS, who were familiar with the entire dataset, provided detailed feedback on CA’s draft analysis, for verification of findings. Participants’ comments were not sought on either the transcripts or study findings. This was impractical because of the end of the college’s academic year and study timelines. We also had concerns for participants’ privacy if we contacted them about the study, given the eligibility criteria and sensitive content of the interviews.

## Results

### Participants

Twenty-five interviews took place in Spring/Summer 2012 (Table 1). Interviewees were aged 16–23 years (mean: 19 years). The quota of 6–8 participants in each sex/age-group category was not filled for older females (*n* = 2 participants) prior to the end of the college’s term.^1^ However CA and (independently) MS, SF, LJS considered saturation to have been reached within the total sample achieved (i.e. no new findings emerged in later interviews). Due to the way recruitment took place, the number approached who declined participation (and their reasons) were not recorded. Two students scheduled an interview but did not participate.

**Table 1:** Participants’ characteristics

Characteristic		Number	
Asked by the interviewer before the interview:			
	Gender	Female	Male
Age^a^	16–17	3	2
	18–19	4	6
	20–21	2	4
	22–23	0	4
Ethnicity^b^	Black/Black British, African	10	
	Black/Black British, Caribbean	6	
	Black British	5	
	Mixed	3	
	Muslim/Asian	1	
Self-defined sexual orientation^b, c^	Straight	22	
	Bisexual	2	
	Gay	1	
Current sexual partner/s	Yes	15	
	No	9	
	‘it’s complicated’	1	
Reported during the interview:			
STI testing experience	Yes	22	
	No	3	
Smartphone ownership	Yes, at time of interview	22	
	Not currently, but has had (lost, in repair, broken)	3	
	Never had a smartphone	0	

^a^For sampling purposes, age-groups were 16–19 and 20–24 years, however no participants were aged 24 years

^b^Self-defined by participants. Ethnicity categories were grouped by researchers

^c^All three respondents self-identifying as bisexual or gay were female

Participants’ accounts of their STI testing experience ranged from a single chlamydia screen, to repeated comprehensive testing in sexual health clinics. Use of STI testing in general practice and use of internet-ordered home-sampling for chlamydia were also reported. Two participants, both women, spontaneously mentioned that they had previously been diagnosed with an STI (however this question was not asked of all participants).

### Perceptions of self-testing with online care pathways, in relation to barriers to use of existing sexual healthcare

Barriers to use of existing sexual healthcare discussed by participants were consistent with those identified in the literature (see Introduction). We focus on perceptions of how the proposed intervention might address barriers to testing using existing services.

#### Making access to STI testing quicker, easier and more convenient

Participants described smartphone-enabled self-testing and online care pathways as making access to STI testing and treatment easier and more convenient than existing services. They associated self-testing with having greater control over when and where they could test – which they welcomed.

*…you could be in the bath, be like using the toilet, and be like, let me just get this real quick and do this real quick. It’s… convenient, very convenient. That’s why I like it* (V, young man, 18–19 years old)

Often, participants assumed that the testing device and online care pathway would be easy to use, though some expressed concerns about operating the device or completing lengthy online forms, emphasising the importance of ease of use.

#### ‘Faceless’ sexual healthcare

Some participants described how concerns about being recognised by staff influenced their STI testing behaviour, and some of those with experience of sexual health clinics described embarrassment around giving a sexual history face-to-face. Self-testing and providing information ‘facelessly’ online was advantageous for these participants.

*I would rather that ‘cause there’s not no one in front of me like talking to me or looking at me…* (C, young woman, 18–19 years old)

#### Concealing use of sexual healthcare

Some male participants, in both age-groups, explained how they did not mind others discovering their use of sexual healthcare. However many, including all of the female participants, spoke of wanting to conceal their sexual healthcare use from family and peers as it suggested or revealed possible STI, risky sexual behaviour or that they were sexually-active. This was described as a barrier to using sexual health clinics: participants described how they might seek ‘*discreet places far away from home’* (F, young woman, 16–17 years old), use internet-based home-sampling, or *‘when you get outside you’ve kind of got to look around and make sure no one sees you and then quickly run in there’* (B, young woman, 18–19 years old). Young women expressed particular concern about the conclusions others might draw about their sexual activity.

Participants welcomed the perceived greater ability they would have to conceal their STI testing by using a self-test, although there were concerns about the test device itself being concealable. There was also anxiety around the presence and visibility of electronic evidence of STI testing on the phone, for instance an app installed on the phone. Related to this point, there was great variation in how privately people described keeping their phones: *‘no one’s really going to look at your phone’* (G, young woman, 18–19 years old), versus ‘*youth nowadays, yeah, we always have each other’s phones’* (Y, young man, 20–21 years old).

### Further perceptions about remote self-testing with online care

In this section we describe perceptions about remote self-testing with online care pathways, which informed an understanding of the proposed intervention’s acceptability, and its development.

#### Speed of testing

Participants expressed varied views about the speed of test-operation (in contrast with their universal interest in rapid access to testing and - if positive - treatment). Some expected a result within minutes, reasoning that new technology ought to provide this; *‘everything is fast now’* (M, young man, 18–19 years old). Others reasoned that a rapid test might be less accurate: a tension between their desire for dependable, yet rapid, results. Those who had used internet-based home-sampling, who described valuing avoiding clinic attendance and/or face-to-face consultations, would rather their results arrived faster than from home-sampling services but accepted waiting days or a week. This suggests that trade-offs exist between speed and privacy, and between speed and perceived accuracy.

#### Self-testing with new technology versus professionals testing using established technology

For some participants *‘a result is a result’* (S, young man, 18–19 years old), assumed to be accurate; they reasoned that clinics also tested urine, stored results on a computer, and with such an important purpose, the testing device would have been checked prior to release. Others questioned the accuracy of results from self-tests. Two main sources of doubt were identified: the novel technology and self-operation. Concerning the technology:

*…this is still new. It has still little kinks to be found, little things to be found. Whereas the clinic is established, they are doing it there and then. But the longer it is out, the more confidence I would get in the technology.* (V, young man, 18–19 years old)

Participants often seemed not to have questioned the accuracy of clinic-based tests, until the interviewer asked whether they would trust the new test. Self-operated technology was an issue for this young person:

*…the clinic, doctors, they’re more professional. That’s exactly what [people] would think because that’s what I would think as well but I would still put trust in my phone.* (X, young man, 16–17 years old)

He went on to say *‘I’d rather get it off the doctor, cos your phone could come back inconclusive.’* Even some of those who said they would trust results from self-tests, described repeat-testing or confirming results in clinic as ways to allay concerns about accuracy. Participants explained that the accuracy of results was extremely important:

*…just don't let it go faulty […] That’s the most important thing in the whole wide world* (F, young woman, 16–17 years old).

#### Personal support from healthcare professionals

There was a tension between participants’ preferences for avoiding clinical contact when accessing testing, and a desire, expressed by some, for contact with a healthcare professional if a positive result were received. Often this was related to anxieties which participants explained might not be addressed through an online service:

*…I will be having thoughts running in my head, so I wouldn’t even have time to go through the link [to access treatment] ‘cause I think there would be tension and pressure on me, so, yeah.* (P, young man, 22–23 years old)

A telephone helpline was considered an acceptable way of providing this human support.

*If you have an infection it should give you information but it should also give you like phone numbers that you can call to talk to someone because at the end of the day I see it as, if it’s something on your phone you don’t really wanna read so much. But if you can talk to someone, not a computer, someone real, then you’re most likely to listen.* (H, young woman, 18–19 years old)

#### Legitimacy and credibility

A basis in the NHS and association with medical professionals enhanced the perceived legitimacy of the proposed service

*That it’s part of the NHS? It makes me feel safe, it makes me feel okay, because like NHS are there to help us innit, like they’re there to help, to support us.* (T, young man, 20–21 years old)

For some, however, a basis in the NHS made little difference provided the service was private and confidential.^2^

#### Confidentiality, data security and trust

Participants were told that with the proposed intervention, users would provide registration information prior to testing. The confidential but not anonymous nature of the service was accepted with varying degrees of reluctance, on the basis that the NHS was trusted and personal information was required to provide any necessary treatment, for one’s own benefit. Participants’ views revealed assumptions that data provided to an NHS service were shared across the NHS, *‘the NHS knows so much about you anyway…’* (Y, young man, 20–21 years old).

There was variation in the extent to which participants trusted their smartphones and the internet, with regard to confidentiality. Passwords, assurance that the app/website was secure, and the legitimacy of the service aided trust in data security.

#### Concealing evidence of an STI

Unsurprisingly, participants described the importance of keeping an STI diagnosis secret. However with the proposed intervention, they discussed how not only the results message, but an ‘e-prescription’ and other messages (e.g. text message reminders to collect treatment) could reveal their STI status, if seen by others. Similarly, preferences for treatment access (collection from community pharmacy using an ‘e-prescription’; or received by post) reflected privacy concerns.

*I don’t like going to the [sexual health] clinic and coming out with prescriptions to be honest with you, but pharmacy, that’s what they’re for.* (G, young woman, 18–19 years old, previous STI diagnosis)

Receiving treatment by post was perceived as more convenient, but slower than pharmacy, with implications for privacy dependent on living arrangements:

*…post is alright too, but then again, because I don’t live by myself, I live with my parents. Then, my mum sometimes likes to open my letters* (I, young woman, 20–21 years old)

### Final word

Overall there was enthusiasm for this innovation: *‘Just get it done quicker, just get it out there fast. Cos it sounds good, so it should be out there’* (L, young man, 20–21 years old).

### Implications for development of the proposed intervention

Table 2 presents tentative design recommendations, and recommendations for further work to develop the proposed intervention. For ease of reference to the analysis, the same headings are used as above.

**Table 2:** Recommendations for the development of STI self-testing within online care pathways

Theme	Recommendations for development
Making access to STI testing quicker, easier and more convenient	The amount of information users need to input should be kept to a minimum.^a^ The device should be easy to use.
‘Faceless’ sexual healthcare	Face-to-face contact with health service staff should be minimised.^b^
Concealing use of sexual healthcare	The self-testing device needs to look inconspicuous (size, appearance). The content and sender name of electronic communications (text messages, emails) should make no reference to STI testing or use of sexual healthcare. An app downloaded to the phone may compromise privacy, so alternatives should be explored.
Speed of testing	The test should give results faster than conventional services, but not necessarily very rapidly.^c^
Self-testing with new technology vs. professionals testing using established technology	Accuracy of results is very important. Accuracy is a concern with self-operation of novel testing technology (ways to increase confidence in the accuracy of the device, and minimise wasteful repeat-testing, need further exploration).
Personal support from healthcare professionals	Optional support from a health professional should be available.^d^ Given the concern for privacy and convenience, this could be by telephone.
Legitimacy and credibility	Confidentiality should be assured. It should be clear to users that the service is part of the NHS.
Confidentiality, data security and trust	It should be clear to users that the service is part of the NHS. Passwords, assurances that the system is secure, and legitimacy (above) aid trust in data security.
Concealing evidence of an STI	The design of the device and care pathways should enable users to keep all evidence of STI secret (including: results message, prescription, treatment) Convenience/discretion of postal receipt of treatment was preferred by some, while others preferred the speed and privacy (from household members) of collecting treatment from community pharmacy.

^a^This needs to be balanced with clinical and disease surveillance requirements

^b^Where medically-appropriate for individuals, and preferred. See also ‘Personal support from healthcare professionals’

^c^Diverse views were expressed, with some perceiving a very fast result to be less accurate

^d^The need for a helpline from a clinical perspective had already been established, but this research confirmed its importance to potential users and its role in providing emotional support

## Discussion

### Main findings

A novel proposal for remote online self-testing and treatment for STIs was broadly acceptable to these ethnic-minority young people from a high-prevalence population. In deciding whether to use existing STI testing services, and considering self-testing, participants appeared to balance three main factors: speed, convenience and privacy. Remote self-testing was perceived to maintain privacy by reducing the risk of peers and family members discovering their use of sexual healthcare, through avoiding sexual health clinic attendance, and by avoiding potentially embarrassing face-to-face consultations. By reducing these privacy concerns, and facilitating access to testing, participants expressed that they might be more likely to test, or test more often, if remote self-testing were available.

New privacy concerns with this novel intervention concerned electronic evidence of sexual healthcare use or STI diagnosis visible on their phone, online data security, and postal provision of treatment. Participants described ways they could manage these risks, and how intervention design could assist with this, but some considered risks to online data security inevitable. Enthusiasm about the novel technology contrasted with some participants’ doubts about the accuracy of a novel, rapid, self-operated test, while accuracy of conventional testing was not questioned. Several participants’ discomfort with sexual health consultations contrasted with their anticipated needs if they received a positive result or had particular concerns: to seek personalised support from healthcare professionals. Credibility of remote self-testing and online care, including data security, was enhanced by its association with healthcare professionals and trusted NHS services.

### Strengths and weaknesses of this study

Formative research is particularly important in the development of complex interventions [27], especially in e-health [28]. During development, qualitative research can contribute to an intervention’s success by informing an understanding of user-behaviour [29], particularly relevant for our intervention, which will be used remotely with minimal supervision. As well as informing an understanding of perceptions and acceptability of the proposed novel intervention, we made specific recommendations for its development (Table 2). Several of these were supported (and none were contradicted) by related formative research [31, 32]. However, as this study took place prior to the availability of the STI self-testing device and operational online care pathways, we relied on participants’ ability to understand and engage with the hypothetical, novel intervention. To make it less abstract we chose a study population among whom STI testing was likely to be familiar, and the interviewer showed an animation to help describe the planned intervention. We decided against restricting recruitment to people with previous STI testing experience or STI diagnosis, as we sought to include those who test infrequently or not at all, who may experience more barriers to testing via existing services. Despite the hypothetical topic, interviews gained rich, detailed accounts of perceptions of smartphone-enabled self-testing, and although only two had been treated for an STI, participants also engaged well with the concept of treatment via an ‘e-prescription’. However many interviewees found provision of treatment to partners difficult to engage with, perhaps because this topic was far from their personal experience and particularly abstract (requiring them to imagine a partner and a context in which STI transmission could have occurred, as well as imagining having been diagnosed with an STI following use of the novel self-test). For reasons of data quality we have not presented findings on this topic.

Engagement with target audiences is recognised as an important challenge to e-sexual health interventions [38], which may be aided by incorporating potential users’ views throughout development. The demographic profile of our participants is close to that of those considered at elevated risk of STI, based on their age, ethnicity and recruitment from an urban, deprived population [2]; thus a key target group for provision of STI services, for reasons of equity and public health need. However, men who have sex with men (MSM), another important risk group for STI, were not targeted for recruitment to the current study because in this educational setting, we did not wish to compromise the privacy of those not ‘out’ to their classmates. Recruitment of exclusively non-White participants (Table 1) was unintentional, largely reflecting the location and student population. (Some White students were approached, but declined participation, with reasons unknown.) The sampling quota for women aged 20–24 (6–8 participants) was not filled (*n* = 2), with implications for analysis and interpretation. Our findings suggest a gender difference in the importance of concealing use of sexual healthcare, but this may also be influenced by female participants’ young age profile, compared to male participants. This was the only clear difference between men and women’s expressed views in relation to the study topic (and there were no clear differences between age-groups), but had we achieved a stronger sample we might have been able to explore age-group and gender differences further.

In qualitative research, it is recognised that the interviewer and participants’ shared or different characteristics influence the data (as discussed in [39]). Data quality is not necessarily considered to be compromised by having a non-peer interviewer [40, 41] (e.g. a male interviewing females). In this study it is encouraging that although the same male interviewer conducted all of the interviews on this sensitive topic, interviewees of both genders discussed their views and experience of sexual healthcare use freely, and the two interviewees who disclosed their previous STI diagnoses (without prompting) were both female. We did not seek participants’ comments on the transcripts, which could have increased data quality, however all transcripts were checked against the audio-recordings. Those interviewed, who chose to participate in a study about sexual health, may be particularly comfortable with STI testing and/or sexual healthcare. However some had little experience of testing, and some discussed their dislike of existing services, so it is unclear what effect this may have had on the data. Non-participants’ privacy concerns may be greater than those discussed by participants, who chose to participate in an interview where they discussed sexual healthcare face-to-face.

As explained, we took steps to reduce social desirability bias, but our study’s premise that STIs are a problem, which can possibly be addressed through new services, was evident in information provided to participants. This may have prompted criticism of existing services. However, participants’ views on existing services reflected those identified in the literature [4–8], and all participants expressed both positive and negative views about aspects of the novel intervention, indicating critical engagement.

### Comparison of our findings with the published literature

We know of no other research exploring the acceptability of remote self-testing linked with online care for STIs, as our proposed intervention is unique. However, our intervention does include some elements that have undergone limited evaluation in other studies. Qualitative research with US young women (conducted 2007–08) reported reservations about internet-use in relation to STI testing, including online privacy and data security concerns, and lack of personal support [23], which feature far less in findings reported in similar qualitative research among Canadian young people [25]. Our study echoed similar findings concerning desire for support from healthcare professionals following a positive diagnosis. Although privacy from peers and family was discussed as important by most participants (related to preferring to self-test instead of attending a sexual health clinic, and preferring discreet messaging) online privacy/confidentiality and security provoked fewer concerns. This possibly reflects our sample’s smartphone ownership, and the confidence in the NHS which they described.

Similarly to our findings, other online sexual health services (internet-ordered home-sampling [23]; downloadable laboratory forms for STI testing without face-to-face consultations [24, 25]) have been perceived positively for their convenience and privacy. US clinic-attenders’ views (focus-groups, 2008–09) on rapid home self-tests for STIs include concerns regarding accuracy and self-operation, and non-immediate treatment access [22]. A US survey on the acceptability of home-sampling among sexual minority youth found similar concerns about test accuracy and home self-sampling [42]. Our participants also expressed concerns around accuracy and self-operation, with linkage to treatment perceived positively.

### Meaning and implications of our study

Our findings suggest that remote self-testing and online care pathways, as described here, would be acceptable as a complement to existing STI services, provided that personal support from healthcare professionals is available to those testing remotely, and accuracy concerns are addressed.

In addition to findings from this (and other) formative research, intervention design must also take account what is technically possible, clinical safety, and public health concerns (see Further Research, below). In the development of the proposed intervention, we need to consider that young people may desire to keep secret not only any STI diagnosis/es, but their sexual healthcare use. Regarding ‘evidence’ of sexual healthcare use on users’ smartphones, care needs to be taken regarding name of the sender and wording of text messages, while web-apps (which are not downloaded or installed to users’ phones) are an alternative to native apps, and NHS branding may confer trustworthiness. For speed and privacy from household members, collection of medication via ‘e-prescription’ from community pharmacies may be more suitable than postal treatment in this young population, depending, of course, on the STI and the nature of the recommended treatment.

Innovations in sexual health clinics, e.g. ‘no-talk’ testing with registration/clinical information provided on paper or electronic forms (e.g. touch-screens) [43–45], may already meet some of young people’s access and privacy needs. However our findings suggest that by removing the need to attend sexual health clinics (for many patients), our intervention may overcome further barriers to sexual healthcare use, resulting in earlier detection of STI. Provided users are able to use the care pathway to access treatment promptly, public health benefits would result from decreased STI transmission and decreased complications of long-term infection.

### Unanswered questions and future research

Findings from our study, together with other formative research [31, 32], have informed intervention design. In terms of its accessibility, potential users’ health literacy and use of appropriate terminology are being considered in its development. Building on this programme of research, online care pathways for chlamydia treatment [32] were recently piloted for feasibility, acceptability and preliminary evidence of effectiveness, and qualitative research was conducted with people who used these online pathways, informed by the findings we present here. Development of the rapid testing device is ongoing. 

Future research must continue to explore the acceptability and feasibility of remote self-testing for STIs and online clinical care pathways, among young people and other potential user-groups (such as MSM, other age-groups and ethnicities), and identify barriers and facilitators to implementation, including costs to users and to the health service. Further research could also explore how acceptability varies between different STIs and when testing for multiple infections. A recent exploratory pilot study about the feasibility, acceptability and safety of an online clinical care pathway for chlamydia was conducted, using mixed-methods (articles in preparation). Sampling limitations of the study reported in this article were addressed in this recent research, which will give us greater scope to explore the role of gender and other factors.

## Conclusions

Our research has informed intervention design, and identified concerns that can be addressed, or need to be explored further. By reducing or removing barriers that participating young people associated with conventional STI testing, our findings suggest that this complex intervention may enable earlier detection and treatment of STIs, thus delivering public health benefit through reduced transmission and reduced complications of infections. Remote STI testing may be a useful adjunct to our repertoire of STI services, ideally integrated within online clinical pathways embedded within existing sexual healthcare provision.

## Additional files

Additional file 1: The animation, showing the proposed testing device and online care pathway. (PNG 1579 kb) Additional file 2: Summary of scenarios discussed in the interviews. (DOCX 20 kb)

## Abbreviations

*e*STI^2^: Electronic Self-Testing Instruments for Sexually Transmitted Infection Control, the Research Consortium developing the remote self-test and online care pathways, and which carried out this study
GUM: Genito-urinary medicine
NCSP: England’s National Chlamydia Screening Programme
NHS: The United Kingdom’s National Health Service
STI: Sexually transmitted infection
UK: United Kingdom (of Great Britain and Northern Ireland)
US: United States (of America)

## Footnotes

1 While A-level courses at the college are taken by younger students of both genders, technical/vocational courses at the college are traditionally gendered (e.g. construction, electronics, beauty). The interviewer encountered more male students than female in the 20–24 age-group on the college campus which taught vocational courses. He therefore asked staff at both campuses to direct him to the locations where there were more 20–24 year old females. Unfortunately he only received this information one week before the end of the academic year, which limited his opportunities to recruit. The high level saturation within the sample achieved, and requirements for the interviews to inform the next phase of our research, led to our decision not to recruit further participants when the college re-opened.

2 During the interviews, participants were asked their understanding of ‘confidentiality’. Despite the interviewer’s explanation during consent-taking, several could not define the term, and several misdefined it as confidence, referring to self-confidence or confidence/trust in healthcare. Participants tended to use the broader term ‘private’ to describe how they wanted their information to be held, and services to be. We use the standard definition of confidentiality, rather than how participants used the term.
