# Supplementary figures and images for: Young people’s perceptions of smartphone-enabled self-testing and online care for sexually transmitted infections: qualitative interview study
Source: BMC Public Health. 2016 Sep 13;16(1):974. doi: 10.1186/s12889-016-3648-y (PMC5022229; doi:10.1186/s12889-016-3648-y)

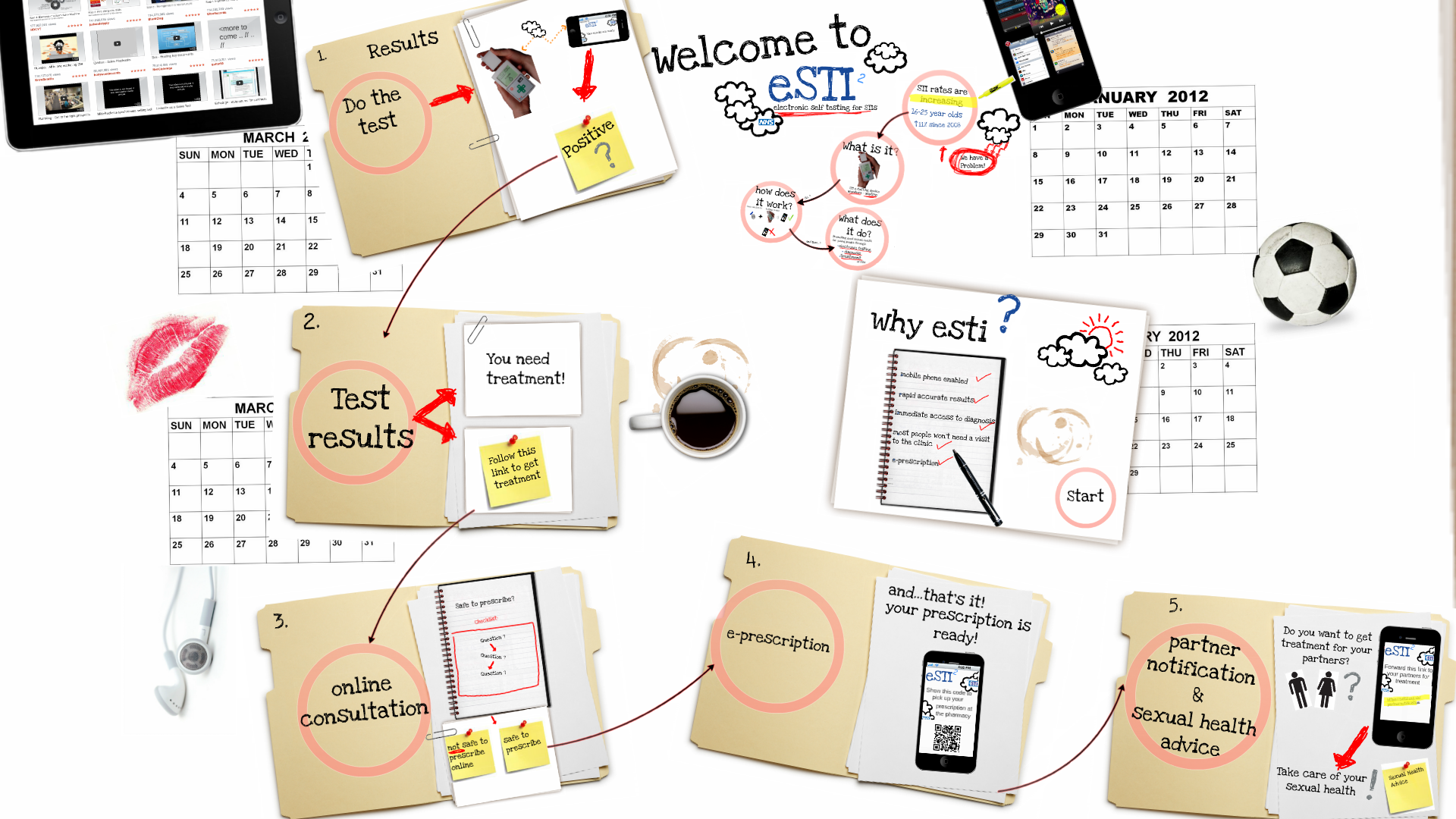

Supplement: Additional file 1: — The animation, showing the proposed testing device and online care pathway. (PNG 1579 kb) [file 12889_2016_3648_MOESM1_ESM.png]
