# Supplementary material for: Young people’s perceptions of smartphone-enabled self-testing and online care for sexually transmitted infections: qualitative interview study
Source: BMC Public Health. 2016 Sep 13;16(1):974. doi: 10.1186/s12889-016-3648-y (PMC5022229; doi:10.1186/s12889-016-3648-y)
Supplement: Additional file 2: — Summary of scenarios discussed in the interviews. (DOCX 20 kb) [file 12889_2016_3648_MOESM2_ESM.docx]

**Additional file 2: Summary of scenarios discussed in the interviews**

*As is usual in qualitative research, the interviewer phrased questions flexibly, with follow-up questions and probes to gain greater depth.*

*These scenarios were explored after discussing initial impressions of ‘self-testing for STIs using your mobile phone’, and after the interviewer had described remote self-testing with online care (aided by the animation). Impressions were revisited at the end of the interview.*

*An additional scenario explored partner notification, not shown here because we do not present results on this topic in this paper.*

| Scenario: |
| --- |
| ACCEPTABILITY OF ONLINE HEALTH PROMOTION ADVICE  If your result was negative (no STI), you would receive a link to online information about sexual health. Would you access this info / read it, or not? (Reasons?) |
| ACCEPTABILITY OF REMOTE ONLINE CARE If your result was positive (you had an STI), you would be given a link so you could get treatment online. Would you do this, or not? (Why/why not? Anything else instead?)  How does getting test results from this new way of testing compare to getting results from a clinic/GP? (Reasons for similarity/difference) |
| ACCEPTABILITY OF INPUTTING INFORMATION VIA SMARTPHONE/COMPUTER, ONLINE  Before using the test, you would be asked for some registration information. This information would be similar to that asked for in clinics, e.g. name, address, number/type of sexual partners. What do you think about providing that information via your mobile phone? (Why? Suggested improvements?)  If you got a positive result (you had an STI) you would be asked for medical information (e.g. allergies, symptoms) to check the treatment is safe for you. Thoughts about providing that information via your mobile phone? (Reasons? Suggested improvements?) |
| ACCEPTABILITY OF REMOTE CARE: TREATMENT  If the standard treatment is safe for you (and for most people it is), you would be sent an electronic prescription. Views on this? Suggested alternatives? |
| TRUST IN REMOTE TESTING/CARE COMPARED TO CLINIC-BASED TESTING/CARE  The ‘e-test’ and ‘e-clinic’ would be part of the NHS so information would be treated the same as in a clinic. Information you provided and STI results would be confidential. Views on this; understanding of confidentiality. |
| REMOTE CARE AS PART OF A ‘VIRTUAL CLINIC’ WITHIN THE NHS  Unlike a pregnancy test bought in a pharmacy, the results you receive from the ‘e-test’ will not be anonymous. That is, you would need to provide registration details to use the service. Views on testing anonymously vs. testing not being anonymous but being able to get an e-prescription for treatment?  After receiving an ‘e-prescription’ you could be sent a text message on your phone, to remind you to pick up and to take your treatment. Views? |
